# Supplementary material for: Single Cell Transcriptomic and Chromatin Profiles Suggest Layer Vb Is the Only Layer With Shared Excitatory Cell Types in the Medial and Lateral Entorhinal Cortex
Source: Front Neural Circuits. 2022 Jan 26;15:806154. doi: 10.3389/fncir.2021.806154 (PMC8826650; doi:10.3389/fncir.2021.806154)
Supplement: Supplementary file 14 [file Data_Sheet_1.ZIP › Inh_vs_Exc_Motifs/homerResults/motif11.similar.html]

motif11

## Information for motif11

T
G
A
C
G
C
T
A
T
C
A
G
T
A
G
C
A
T
C
G
A
G
T
C
G
C
A
T
A
C
T
G
T
A
G
C
G
C
T
A
T
C
A
G
G
T
C
A
  
Reverse Opposite:  

C
A
G
T
A
G
T
C
C
G
A
T
A
T
C
G
T
G
A
C
C
G
T
A
T
C
A
G
T
A
G
C
A
T
C
G
A
G
T
C
C
G
A
T
A
C
T
G
  

|  |  |
| --- | --- |
| p-value: | 1e-17 |
| log p-value: | -4.012e+01 |
| Information Content per bp: | 1.589 |
| Number of Target Sequences with motif | 2438.0 |
| Percentage of Target Sequences with motif | 19.92% |
| Number of Background Sequences with motif | 6530.7 |
| Percentage of Background Sequences with motif | 16.93% |
| Average Position of motif in Targets | 312.8 +/- 250.8bp |
| Average Position of motif in Background | 336.8 +/- 339.9bp |
| Strand Bias (log2 ratio + to - strand density) | 0.1 |
| Multiplicity (# of sites on avg that occur together) | 1.14 |
| Motif File: | file (matrix) reverse opposite |

### Similar de novo motifs found

|  |  |  |  |  |  |  |  |
| --- | --- | --- | --- | --- | --- | --- | --- |
| Rank | Match Score | Redundant Motif | P-value | log P-value | % of Targets | % of Background | Motif file |
| 1 | 0.797 | C G T A T C A G A T G C A T C G T A G C G C A T A T C G T A G C | 1e-11 | -26.551066 | 10.85% | 9.01% | motif file (matrix) |
